# Prevalence of Early Childhood Caries in Southern Italy: An Epidemiological Study

**DOI:** 10.1155/2021/5106473

**Published:** 2021-05-25

**Authors:** Grazia Fichera, Simona Santonocito, Vincenzo Ronsivalle, Alessandro Polizzi, Salvatore Torrisi, Ludovica Deodato, Giuseppe Palazzo, Gaetano Isola

**Affiliations:** ^1^Department of General Surgery and Surgical-Medical Specialties, School of Dentistry, University of Catania, Via S. Sofia 78, Catania 95124, Italy; ^2^Department of Biomedical and Dental Sciences and Morphofunctional Imaging, Section of Orthodontics, University of Messina, Via Consolare Valeria 1, Messina 98123, Italy

## Abstract

The purpose of this study was to assess the prevalence of Early Childhood Caries (ECC) and oral habits among preschool children from a southern Italian cohort. The survey population consisted of 300 subjects randomly selected among children attending two kindergartens in Catania, Italy. The prevalence of ECC and oral habits were clinically evaluated. During the clinical evaluation, the oral hygiene of children was also assessed. Parents were also interviewed using a brief and simple online questionnaire aimed at investigating eating and oral hygiene habits. The selected sample age ranged between 3 and 5 years. The prevalence of ECC was 9.34%, and the most affected teeth were primary molars. Moreover, the prevalence of oral habits was 44.66%. Only two-thirds of the sample brush their teeth at least once per day. The prevalence of ECC among Sicilian children (9.33%) seems suitable with the rest of the country. The results of the present study evidenced that oral habits showed a high prevalence among preschool children. The level of oral hygiene among preschool children is still not sufficient.

## 1. Introduction

It has been widely demonstrated that good oral health and quality of life are very important for human well-being. The World Health Organization (WHO) defines oral health as “a state of being free from mouth and facial pain, oral and throat cancer, oral infection and sores, periodontal (gum) disease, tooth decay, tooth loss, other diseases and disorders that limit an individual's capacity in biting, chewing, smiling, speaking, and psychosocial well-being” [2, 3].

Among oral affections, Early Childhood Caries (ECC) is one of the most common forms of oral diseases in childhood [4]. The ECC is defined as a carious disease that develops in children under the age of 6 [5]. It is a multifactorial pathology whose main cause is to be found in the prolonged consumption of sugar, honey, and sugary drinks, but also milk without sugar added (especially at night, when the salivary flow is strongly reduced) administered to the very young child through pacifier or bottle. In children younger than 3 years of age, any sign of smooth-surface caries is indicative of severe ECC. The clinical picture varies from the initial forms of demineralization of the enamel to the complete amputation of the milk teeth. According to the current literature, the prevalence of ECC differs among studies, with a prevalence among preschool children (≤6 years old) in a range of 12% to 98% [4, 6, 7]. Recent studies have shown that, in children between 2 and 6 years of age, caries were found in 40% of children in a cohort of the United States, while in a range of 10% in a study on Dutch children, 3-year-old [9]. Furthermore, some others studies demonstrated a wide range of pre-carious and white spot lesions on 26% of the patients in Qatar [4, 10].

ECC is a biofilm (plaque)-induced acid disease with a multifactorial aetiology which determines demineralization of enamel or dentin, mediated by saliva, and is highly influenced by socioeconomic status (SES) and education of children and their caregivers [11, 12]. ECC is diagnosed by the presence of 1 or more decayed (noncavitated or cavitated lesions), missing (due to caries), or filled tooth surfaces in any tooth in a child 71 months of age or younger. In children younger than 3 years of age, any sign of smooth-surface caries indicates severe ECC [12]. Moreover, for ECC development, parents' attitudes and practices have been shown to be important, including diet and oral hygiene practices undertaken with their children [13].

ECC is determined by the teeth structure characteristics (absence of exposure to fluorine, defect of enamel or genetic factor), as well as the oral colonization of cariogenic bacteria (due to saliva composition, poor oral hygiene, and mother's health) [14, 15], diet (high sugar diet, feeding practices, and nocturnal breastfeeding) [16], and environmental factors (low birth or premature birth weight, poor education of parents with low socioeconomic status) [15, 17]. An important risk factor is sugars consumption in the diet [18]. In this regard, the WHO recommends limiting sugar intake to limit the incidence of caries, particularly those added in food and beverages, and limiting foods with natural sugars such as honey or fruit juices, while milk sugars are excluded. In general, it is recommended to take a maximum of 10% of the total daily calories of sugars for both adults and children [17].

Moreover, also oral habits, such as oral breathing, atypical swallowing, or non-nutritive sucking, can widely affect oral health, interfering with dental occlusion and craniofacial development [19–21]. Thus, primary prevention is necessary in the early phase of childhood to reduce the risk of caries initiation and to avoid its further development. The preschool period is the time in which deleterious oral habits, caries patterns, and risk factors are established. Those habits have a reported prevalence that ranges around 30–80% [22]. Poor oral health significantly affects children's nutritional intake and consequently their general health, growth and development, imposing considerable financial, social, and personal burdens [23, 24].

In the early stages of ECC, often there are no symptoms, but later, the progress of the lesion involves a slight pain and discomfort [17]. If the ECC is not treated, it can cause difficulties in eating, sleeping, talking, and hindering the normal growth of the child [25, 26]. It has been suggested that children who suffer from ECC have lower body weight and height than those without ECC [27], requesting, in some serious cases, a need for emergency dental visits or the need for hospitalizations [28, 29].

According to the current literature, the prevalence of ECC varies widely among populations and, within the same population with uncertain results. The present cross-sectional epidemiological study aimed to investigate ECC, oral habits, and oral hygiene in a cohort of preschool children from southern Italy.

## 2. Materials and Methods

The present study followed the Helsinki Declaration on medical protocols. The local International Review Board of the University of Catania approved the study protocol (protocol *n*. 17/19). The study was performed in accordance with a regional health protocol for ensuring orthodontic and oral screening among children. For this study, a sample of children was randomly selected from two kindergartens (Catania, Italy) between May and December 2019 which adhered to a healthy regional protocol for screening oral health status. The kindergartens considered are inside the city centre, where users belong to a middle social class, analyzed through the assessment of their Socioeconomic Status (SES). The criteria for inclusion of the study were to attend kindergarten and the absence of systemic pathologies. All the subjects were screened in their classrooms with the respective teachers' presence to keep them in a familiar environment achieving the maximum cooperation from them. The enrolled subjects were randomly assigned to the study using a computer-generated table.

Standardized methods for clinical evaluation were used to examine the subjects. The oral screening was carried on by one trained operator using a kit composed by a mouth mirror, a dental probe, and a portable light source. The Decayed, Missing, and Filled Teeth (DMFT) index was used to assess the state of caries; the oral hygiene conditions and the presence of oral habits (such as oral breathing, atypical swallowing, non-nutritive sucking, or incorrect postural attitudes) were detected by anamnestic and clinical examination.

Parents were also interviewed using a brief and simple online questionnaire specifically created for the purpose of the present investigation. Data were collected on selected lifestyle habits of the parents potentially affecting their children's oral health, particularly the usage of pacifier, baby bottle, and snacks. Parents were also asked to report if they ever brought their children to a dental specialist and the reason.

### 2.1. Statistical Analysis

The sample size was established considering an effect size of 0.30 for decayed teeth (that represented the primary outcome variable), a two-sided significance level of 0.05, and a power of 80%. The data obtained from each examined subject were collected using a predefined clinical and oral chart. Instead, in the presence of specific variables, in order to analyze data, the Chi-square test was used for inferential statistical evaluations. The odds ratio was also used to investigate the risk of ECC occurrence given the exposure to specific variables. Two independent clinicians performed the clinical examination and the diagnostic agreement was evaluated after data collection by using the Cohens' Kappa coefficient.

## 3. Results

For this study, a sample of 300 children, 148 males and 152 females, aged between 3 and 5 years (average 3.7 ± 1.1 of age).  Table 1 shows descriptive data as well as data from parents' interview. There was an increased risk of caries associated with taking snacks between meals (Table 1). Moreover, pacifier with sugared substances was associated with ECC (OR 2.90). As summarized in Table 2, of the total sample of 300 subjects, 272 were dental caries-free, 28 of them had dental caries experience (16 males, 12 females), with a prevalence of 9.34%, only 6 of those were undergoing any treatment for dental caries, while the DFMT index was 0.44 (Table 3). The distribution of dental caries among teeth is reported in Table 4; 94% of decayed, extracted, or filled teeth were primary molar teeth and only 3% were incisor or canine. Oral habits such as oral breathing, atypical swallowing, non-nutritional sucking, and incorrect postural attitudes were reported in 134 subjects, with a total prevalence of 44.66% (Table 5). Moreover, 66.66% of the subjects brushed their teeth at least once a day alone and/or with the help of their parents (Table 6). The inter-rater agreement for clinical assessment of ECC and DFMT index was 0.983 and 0.965, respectively.

## 4. Discussion

This study aimed to assess the prevalence of ECC and oral habits among preschool children from a southern Italian cohort. The present study results evidenced that oral habits showed a high prevalence among preschool children, with a significant role played by SES and education in the analyzed sample.

In this regard, current literature has confirmed the important role of SES and education on the prevalence and prevention of ECC; these factors may also influence the possibility of an early diagnosis of oral habits, which can lead to worsen children's oral health [1]. Consequently, it has been shown that it is important to assess and control the prevalence of ECC and oral habits in a wide range of young populations worldwide [2–5]. From the latest results in the literature, this is among the few surveys that specifically investigated the prevalence of ECC and oral habits among preschool children from Sicily, Italy.

In this regard, although the Italian Clinical Recommendations in Odontostomatology [2] indicates 18 to 24 months as the best time period for the first dental visit, however it should be underlined that over 60% of children aged 24 months had never received dental examination [3, 4], still representing a major concern that suggests the necessity to promote early dental visits in children through large scale population programs [5]. The present investigation also confirms this since only 29% of the investigated subjects underwent dental visit, with paediatricians' referrals being the first reason to seek dental specialists.

In low-income countries, the total prevalence of ECC is as high as 70%, while in countries with high incomes, a prevalence of less than 20% is reported [1, 4]. In Europe, ECC prevalence studies are carried out on children aged 3 and over, mainly on samples of kindergartens or primary school children [6, 7]. These surveys provide a current prevalence of ECC, given the great relationship between age prevalence and caries. In comparison, some studies in Europe have demonstrated that the ECC prevalence was 12% in England among 3-year-old children, 23% in Switzerland among children aged 3–5 years [6, 8], and 11% in Sweden among 3–6-year-old children [7]. Regarding the ECC prevalence in Italy, Campus et al. [9] found a 22% prevalence of ECC on a sample of 5.538 4-year-old Italian children, while Nobile in 2014 on children from southern Italy between 3 and 5 years of age showed a prevalence of ECC in the 19% of the population analyzed [10]. Similarly, a study conducted by Ferro et al. on a sample of 2603 children from both northern, central, and southern Italy showed a prevalence of ECC in children aged between 3 and 5 years of 17%, 24%, and 35%, respectively. [11]. In comparison, the present study results evidenced an ECC prevalence of 9.3% among children, 3–5 years old, significantly different from those than previous Italian studies [9–11]. Furthermore, although a high percentage of children in the sample did not practice adequate oral hygiene habits, the low incidence of ECC may also have been affected by a low-sugar Mediterranean diet.

The present study results evidenced a prevalence of ECC in the screened southern population comparable with the data reported in previous studies conducted in some northern Italian populations [3]. More specifically, the teeth mostly affected from dental caries were primary molars, in agreement with the current literature [12]. The consistency of the clinical data collection of ECC and their clinical relevance were confirmed by the high agreement found between two different clinicians.

Bearing in mind the detrimental functional consequence of ECC, it must be underlined that the early loss of posterior dentition can also compromise the possibility of performing orthodontic protocols in mixed dentition such as maxillary skeletal expansion or orthopaedic/functional therapies [13, 14]. This occurs since the absence of healthy posterior teeth does not provide the appropriate anchorage for the possible future therapy by fixed orthodontic appliances or compels the usage of more invasive devices such as those supported by skeletal anchorage [8, 15].

The prevalence of oral habits among screened subjects was 44.6%, unfolding the relevance of this phenomenon and the poor knowledge among children's caregivers. Considering the potential consequences of oral habits, prevention procedures should be taken into account, involving parents, pediatric dentists, and paediatricians [16, 17].

Furthermore, the results of the present study evidenced that, in the present cohort, there was an increased risk of caries associated with snack assumption between meals and sugared substances. This should be underlined in order to instruct both children and caregivers on a correct diet and the relative creation of specific oral hygiene programs.

With regard to oral hygiene habits, the results indicate that oral health education knowledge is beginning to find resonance among caregivers and school staff, but considering that only two-thirds of the screened children reported to brush their teeth at least once per day, greater efforts are needed to improve the knowledge and practice of good oral hygiene procedure [18, 19]. In this respect, previous evidence confirmed that good oral hygiene reduces the risk of dental caries [8, 20, 21]. Considering the mean age of the subjects included in the present study, specific recommendations can be drafted, such as the need for tooth brushing at least once per day, preferably at bedtime, and giving the child “ownership” of brushing in the morning, depending on age and responsibility level.

In this regard, parents' rule is fundamental since it must provide a leading guide to good oral hygiene behaviour of their children. However, there is also a direct causative relation between parents' personal oral hygiene behaviour and the oral health condition of their children. For instance, it has been shown that high number of caries of the parents and low frequency of tooth brushing augment the risk of ECC in their children [22, 23]. This is due to the transmission of the *Streptococcus mutans* being significantly augmented when inappropriate behaviors [24–26] are pursued such as the usage of the baby bottle, the pacifier, or the same cutlery not clean shared with the parent's saliva [3, 27–32]. In this respect, the mother can be an important font of transmission in children especially in the first two years of life, when *Streptococci mutans* are initially transferred, especially using dental materials [3, 33–36]. Thus, if the mother does not follow appropriate behaviour of oral hygiene [27–31], the risk of development of cavities in the child increases since the concentration of bacteria is higher and more aggressive [37–40].

In conclusion, the present results suggest that it is important to organize and bring specific regional and national oral health campaigns to inform parents of simple good practices aimed at early prevent caries in children, given the dangerous consequences of the ECC in the long term. Moreover, concerning oral prevention, paediatricians are called to play a crucial role since they can first identify the oral disease and inform parents on the good practices of oral disease prevention.

However, the present study has some limitations that need to be addressed, mainly linked to the limited sample size analyzed. Moreover, the absence of intra-observer agreement among clinicians may have affected some study data.

## 5. Conclusions

In the last few decades, several studies have analyzed the prevalence and impact of ECC on some populations. The present study evidenced that there was a mild ECC prevalence among the southern Italian population of children that was analzyed. Moreover, oral habits showed a high prevalence among preschool children, while oral hygiene among preschool children is still insufficient. However, the present study results are promising and demand further larger studies, maybe with a multicenter and more longer prospective design to better analyze the prevalence of ECC and oral habit.

## Figures and Tables

**Table 1:** Descriptive data of the sample study and odd ratio for early child caries [1].

		Sample	Prevalence	ECC
		*N*	%	OR (95% C.I.)
Total		300	—	
*Sex*	Male	148	49.33	0.84 (0.69–1.06)
	Female	152	50.66	

*Average age*		3,7 ± 0.6	—	—

*Baby bottle with milk*	Yes	29	9.6	1.65 (1.33–1.97)
	No	271	91.4	

*Pacifier with sugared substances*	Yes	8	2.66	2.90 (2.44–3.37)
	No	292	97.44	

*Snacks between meals*	No	49	16.33	—
	<2	153	51	0.91 (0.61–1.22)
	±2	98	32.66	1.92 (1.46–2.39)

*Visit to the dentist*	Yes	87	29	—
	No	213	71	

*Reason for visit in dental clinic*	Pain	5	5.64	—
	Trauma	7	8.04	
	Pediatrician	66	75.66	
	Other specialists	9	10.34	

**Table 2:** Prevalence of dental caries.

	*N*	%
Subjects free from dental caries	272	90.66
Subjects with untreated dental caries	22	7.33
Subject with filled or extracted decayed teeth	2	0.66
Subjects with filled teeth	2	0.66
Subjects with extracted teeth	2	0.66

**Table 3:** DFMT index.

	Teeth	DFM
Decayed	122	0.02
Missing	6	0.02
Filled	6	0.02
Total	134	0.44

**Table 4:** Distribution of dental caries, extraction, or filling among primary teeth.

	Decayed, *n* (%)	Extracted, *n* (%)	Filled, *n* (%)	Total, *n* (%)
Incisor	4 (7.46%)	0	0	4 (3%)
Canine	4 (7.46%)	0	0	4 (3%)
Molars	114 (85.08%)	6 (4.47%)	6 (4.47%)	126 (94%)

**Table 5:** Prevalence and distribution of oral habits.

	*n*	%
Oral breathing	18	6
Atypical swallowing	44	14.66
Non-nutritional sucking	52	17.33
Incorrect postural attitudes	20	6.66
Total	**134**	**44.66**

**Table 6:** Frequency of daily teeth brushing.

Teeth brushing	*n*	%
1 time a day	116	38.66
2 times a day	52	17.3
3 times a day	32	10
Rarely	82	27.33
Never	18	6

## Data Availability

The data used to support the findings of this study are freely available from the corresponding author on reasonable request.
